# Hepatitis B Virus Pre-S2 Mutant Induces Aerobic Glycolysis through Mammalian Target of Rapamycin Signal Cascade

**DOI:** 10.1371/journal.pone.0122373

**Published:** 2015-04-24

**Authors:** Chiao-Fang Teng, Wen-Chuan Hsieh, Han-Chieh Wu, Yih-Jyh Lin, Hung-Wen Tsai, Wenya Huang, Ih-Jen Su

**Affiliations:** 1 National Institute of Infectious Diseases and Vaccinology, National Health Research Institutes, Tainan, Taiwan; 2 Department of Surgery, National Cheng Kung University Hospital, Tainan, Taiwan; 3 Department of Pathology, National Cheng Kung University Hospital, Tainan, Taiwan; 4 Department of Medical Laboratory Science and Biotechnology, National Cheng Kung University College of Medicine, Tainan, Taiwan; University of Medicine, Greifswald, Germany, GERMANY

## Abstract

Hepatitis B virus (HBV) pre-S2 mutant can induce hepatocellular carcinoma (HCC) via the induction of endoplasmic reticulum stress to activate mammalian target of rapamycin (MTOR) signaling. The association of metabolic syndrome with HBV-related HCC raises the possibility that pre-S2 mutant-induced MTOR activation may drive the development of metabolic disorders to promote tumorigenesis in chronic HBV infection. To address this issue, glucose metabolism and gene expression profiles were analyzed in transgenic mice livers harboring pre-S2 mutant and in an *in vitro* culture system. The pre-S2 mutant transgenic HCCs showed glycogen depletion. The pre-S2 mutant initiated an MTOR-dependent glycolytic pathway, involving the eukaryotic translation initiation factor 4E binding protein 1 (EIF4EBP1), Yin Yang 1 (YY1), and myelocytomatosis oncogene (MYC) to activate the solute carrier family 2 (facilitated glucose transporter), member 1 (SLC2A1), contributing to aberrant glucose uptake and lactate production at the advanced stage of pre-S2 mutant transgenic tumorigenesis. Such a glycolysis-associated MTOR signal cascade was validated in human HBV-related HCC tissues and shown to mediate the inhibitory effect of a model of combined resveratrol and silymarin product on tumor growth. Our results provide the mechanism of pre-S2 mutant-induced MTOR activation in the metabolic switch in HBV tumorigenesis. Chemoprevention can be designed along this line to prevent HCC development in high-risk HBV carriers.

## Introduction

Epidemiological studies have provided overwhelming evidence for a causal role of chronic hepatitis B virus (HBV) infection in the development of human hepatocellular carcinoma (HCC) [1]. Although several mechanisms have been proposed to explain HBV-related tumorigenesis [2,3], the pathogenesis of HBV carcinogenesis is still elusive. Previously, we demonstrated that HBV pre-S2 mutant identified in type II ground glass hepatocytes (GGHs) can induce endoplasmic reticulum (ER) stress and oxidative DNA damage, as well as exhibits transforming capabilities [4]. Transgenic mice harboring pre-S2 mutant can induce nodular dysplasia and HCC [5]. Moreover, subsequent studies have revealed the positive predictive value of pre-S2 mutant and type II GGHs in HCC development [6–8]. Therefore, type II GGHs represent preneoplastic lesions of HBV-related HCC, and pre-S2 mutant is now recognized as a potential viral oncoprotein [9,10].

Metabolic changes are common features in the development of many types of human cancers [11]. Reports have established that cancer cells frequently display high rates of aerobic glycolysis in comparison to their nontransformed counterparts, a phenomenon known as the “Warburg effect”, to support the increased demand of macromolecules for cell growth and proliferation [12]. Recently, numerous reports have uncovered multiple metabolic changes in HCC, among which elevated glycolysis is one of the principal changes linked to highly proliferative malignant phenotype [13–15]. Previous study based on HBV transgenic mice has also consistently revealed a metabolic alteration of hepatocytes from the glycogen-storage (glycogenotic) state toward an increase of glycolysis (the glycogen-poor state) during neoplastic transformation [16]. However, the underlying mechanism of HBV in aerobic glycolysis in HCC development remains to be clarified.

The mammalian target of rapamycin (MTOR) is a highly conserved serine/threonine kinase that controls cell growth and proliferation [17]. In addition to its better-known functions in promoting protein synthesis, MTOR is now emerging as a key regulator of cellular metabolism and cancer [18]. Research has documented that MTOR activation is sufficient to stimulate specific metabolic pathways, including aerobic glycolysis [19]. Previously, we have demonstrated that HBV pre-S2 mutant can activate MTOR through the induction of ER stress-dependent vascular endothelial growth factor A (VEGFA)/AKT signaling in GGHs to promote tumorigenesis [20]. The activated MTOR signal can further upregulate the Yin Yang 1 (YY1) [21], a transcription factor involved in cell proliferation and regulation of oncogenes [22]. This study was designed to investigate whether pre-S2 mutant-induced MTOR activation may regulate aerobic glycolysis through YY1 signaling cascade in HBV-related tumorigenesis.

## Materials and Methods

### Transgenic Mice

The transgenic mice expressing HBV pre-S2 mutant and X proteins in liver were established by Professor Ting-Fen Tsai’s laboratory as described [23]. All animal experiments were performed in male mice after sacrifice by CO2 inhalation under the approval of the institutional animal care and use committees of the National Cheng Kung University College of Medicine and the National Health Research Institutes.

### Histopathology, Immunohistochemistry (IHC), and Immuofluorescence (IF) Studies

For histopathological examination, paraffin-embedded liver sections were stained with hematoxylin-eosin (HE). IHC and IF staining were described in the previous report [5]. The primary antibodies used in this study were anti-HBsAg (HBV surface antigen) (Dako, San Ramon, CA), anti-SLC2A1 (Proteintech Group, Chicago, IL), and anti-HA-tag (Santa Cruz Biotechnology, Santa Cruz, CA). Glycogen was visualized with periodic acid-Schiff (PAS) staining (Muto Pure Chemicals, Tokyo, Japan) following the manufacturer’s instructions.

### cDNA Microarray Analysis

The microarray experiments were performed by Welgene Biotech (Taipei, Taiwan) using the Agilent Mouse Whole Genome (4×44K) Oligo Microarray Chips (Agilent Technologies, Santa Clara, CA). All values were shown as fold changes relative to the data of age-matched non-transgenic livers. The ≥1.5-fold changes at each stage were considered as significant upregulation or downregulation.

### Real-Time Polymerase Chain Reaction (PCR)

Total RNA was extracted and converted to complementary DNA as described [21]. Real-time PCR was performed with the LightCycler system and Mouse Universal ProbeLibrary system (Roche Applied Science, Indianapolis, IN). The primers and probes used are shown in S1 Table.

### Plasmid, Cell Line, and Transient Transfection

The plasmids pIRES-Δ2 and pIRES-X expressing HA-tagged HBV pre-S2 mutant and X proteins, respectively, were established as described [5]. Rapamycin was purchased from Calbiochem (San Diego, CA) and used at a final concentration of 100 nM. All siRNAs were obtained as ON-TARGETplus SMARTpool reagents (a mixture of 4 individual siRNAs) (Dharmacon, Lafayette, CO) and used at a final concentration of 50 nM. The human hepatoma cells (HuH-7 and HepG2) were obtained from the Health Science Research Resources Bank (JCRB0403; Osaka, Japan) and the American Type Culture Collection (ATCC HB-8065; Rockville, MD), respectively. All transfections were performed with the MicroPorator (Invitrogen Life Technologies, Carlsbad, CA) following the manufacturer’s instructions. Cell proliferation was measured by MTT assay using the Cell Counting Kit-8 (Sigma, St. Louis, MO) following the manufacturer’s instructions.

### Western Blot Analysis

Western blot analysis was performed as described.[21] The primary antibodies used in this study were anti-pMTOR (Ser2448), anti-E2F2, anti-SLC2A2, anti-SLC2A3, and anti-SLC2A4 (Abcam, Cambridge, UK), anti-YY1, and anti-CCNA2 (Santa Cruz Biotechnology), anti-MYC, anti-CCNE1, anti-EIF4EBP1, anti-pEIF4EBP1 (Thr37/46), anti-RPS6KB1, and anti-pRPS6KB1 (Thr389) (Cell Signaling Technology, Danvers, MA), anti-SLC2A1 (Novus Biologicals, Littleton, CO), anti-HA-tag (Zymed Laboratories, South San Francisco, CA), and anti-ACTB (actin, beta) (Chemicon, Temecula, CA). The plasma membrane proteins were prepared with the Qproteome Plasma Membrane Protein Kit (Qiagen, Valencia, CA) following the manufacturer’s instructions.

### Glycogen, Glucose, and Lactate Measurements

Glycogen levels in liver tissues were determined using the Glycogen Colorimetric/Fluorometric Assay Kit (Biovision, Mountain View, CA) following the manufacturer’s instructions. Glucose uptake and lactate production are reported as glucose utilization and lactate secretion per cell within the observed period, respectively. To that end, glucose and lactate concentrations were measured in supernatants using the Glucose (HK) Assay Kit (Sigma) and Lactate Assay Kit (Sigma) following the manufacturer’s instructions, respectively. Each experiment was performed in triplicate and repeated at least three times independently.

### Human HCC Liver Tissues

Decoding freshly frozen human HBV-related HCC tissues were obtained from the Human Biobank, Research Center of Clinical Medicine, National Cheng Kung University Hospital, Tainan, Taiwan (URL: http://tissuebank.med.ncku.edu.tw), under the approval of the National Cheng Kung University Hospital Institutional Review Board (A-BR-101-133). The National Cheng Kung University Hospital Institutional Review Board waived the need for informed consent.

### The model of combined resveratrol and silymarin product on HCC growth

Resveratrol (0.875 mg Vineatrol 30/g chow) (Breko GmbH, Bremen, German) and silymarin (0.875 mg Silymarin/g chow) (Indena, Milan, Italy) were mixed with normal chow diets and fed to the transgenic mice at the age of 8 months for 6 months and then sacrificed. The transgenic mice livers were sampled for analyzing the gene expression profiles.

### Statistical Analysis

The significance of selected biomarkers in transgenic mice and human livers was determined by unpaired and paired t tests, respectively. The *in vitro* data were analyzed by one-way ANOVA with Bonferroni’s multiple-comparison post-test. A *P* value of<0.05 was considered statistically significant (* *P* value<0.05; ** *P* value<0.01; *** *P* value<0.001). The data represent the mean with standard deviation error bar.

## Results

### Pre-S2 mutant transgenic mice exhibited glycogen depletion in HCC tissues

**Fig 1:**
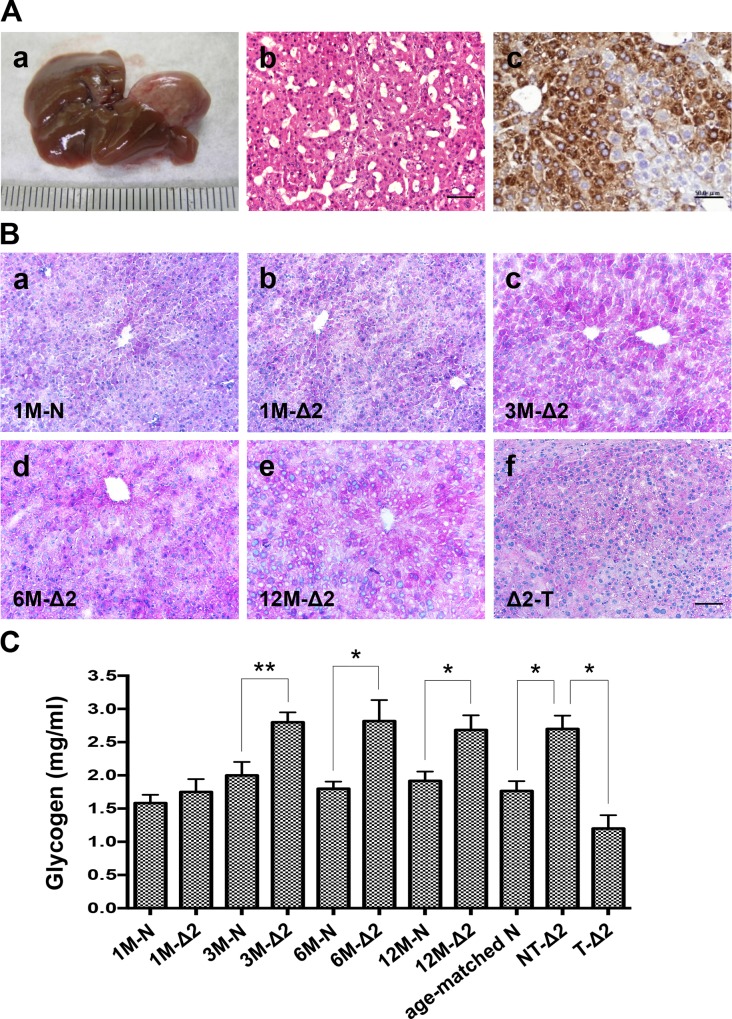
Pre-S2 mutant transgenic mice exhibited glycogen depletion in HCC tissues: (A) Gross view of representative HCC (a), histological HE staining of tumor (b), and immunohistochemical detection of HBsAg (c). (B) PAS staining for glycogen. (C) Glycogen levels in mice livers. Shown were representative results from different months (M) of pre-S2 mutant (Δ2) and non-transgenic (N) livers, nontumorous livers (NT), and tumors (T). Original magnification, ×20. Scale bar, 50 μm.

In the pre-S2 mutant transgenic mice model, male mice developed HCCs at the mean age of 24.5 months with a 12% occurrence rate and expressed HBsAg in the hepatocytes around the typical central vein (Fig. 1A). The transgenic livers were sampled for histopathologic studies at 1 and 3 months (early stage), 6 months (middle stage), 12 months (advanced stage), and at tumor formation. PAS staining showed that the livers contained high glycogen contents in 3-, 6-, and 12-month-old transgenic mice, but not in 1-month-old transgenic mice, as compared with the age-matched non-transgenic mice (Fig. 1Ba,b,c,d,e). Interestingly, when the transgenic livers progressed to HCCs, the glycogen staining became slightly weaker and tended to be scattered and mixed with the vacuoles (Fig. 1Bf). The amount of glycogen in mice livers was further determined and showed a significant decrease in transgenic HCCs (Fig. 1C).

### MTOR, YY1, MYC, and SLC2A1 signals were chronologically activated in pre-S2 mutant transgenic livers and HCCs

**Fig 2:**
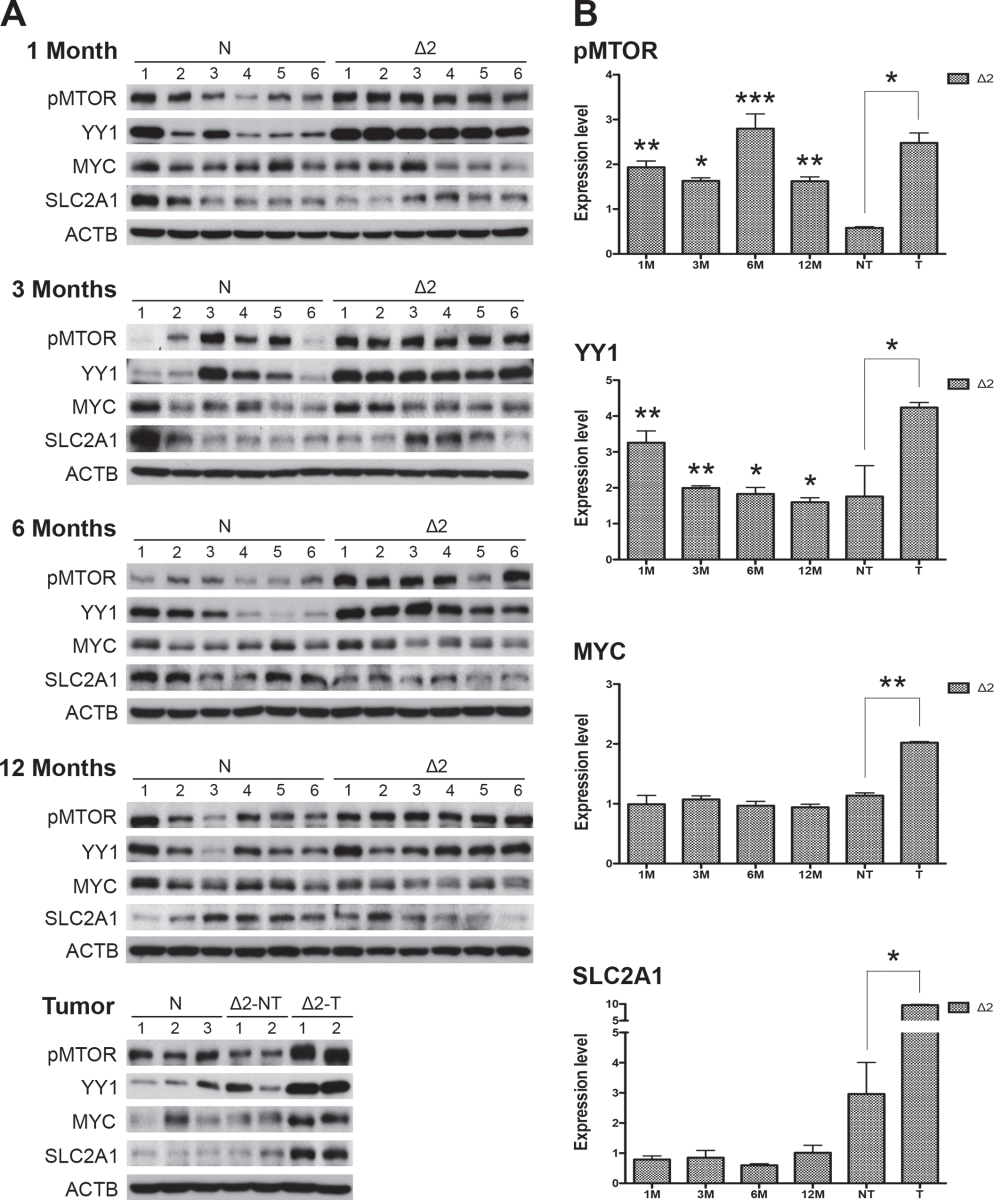
MTOR, YY1, MYC, and SLC2A1 signals were chronologically activated in pre-S2 mutant transgenic livers and HCCs: (A) Western blot analysis of the indicated biomarkers in different ages of pre-S2 mutant and non-transgenic livers, as well as paired nontumorous livers and tumors. Six livers were used in each group except the tumor stage due to small tumor size and low tumor formation rate. (B) Quantitative results were normalized by age-matched control livers.

The cDNA microarray data of pre-S2 mutant transgenic livers was adopted to identify the YY1-activated oncogenes. *Myc* was identified as the only gene showing significant upregulation (≥1.5-fold) in transgenic HCCs relative to the non-transgenic livers (S1A Fig.). By further analysis of the microarray data for potential MYC-activated glycolytic genes, we found that *Slc2a1* expression was significantly increased in transgenic HCCs (S1B Fig.). The increased levels of *Myc* and *Slc2a1* transcripts in transgenic HCCs were further confirmed by real-time PCR (S1C Fig.). These observations led us to speculate whether pre-S2 mutant might regulate tumor glycolysis through activation of the MTOR/YY1/MYC/SLC2A1 signaling cascade. To test this hypothesis, Western blot analysis was performed to evaluate the expression of genes involved in the proposed signaling pathway. We observed that the expression levels of the phosphorylated active form of MTOR (pMTOR) and YY1 were significantly elevated at as early as one month of age and persistently activated throughout the study period, while MYC and SLC2A1 expression was found upregulated only upon tumor formation (Fig. 2).

### Pre-S2 mutant activated MTOR/YY1/MYC/SLC2A1 signaling cascade in HuH-7 cells

**Fig 3:**
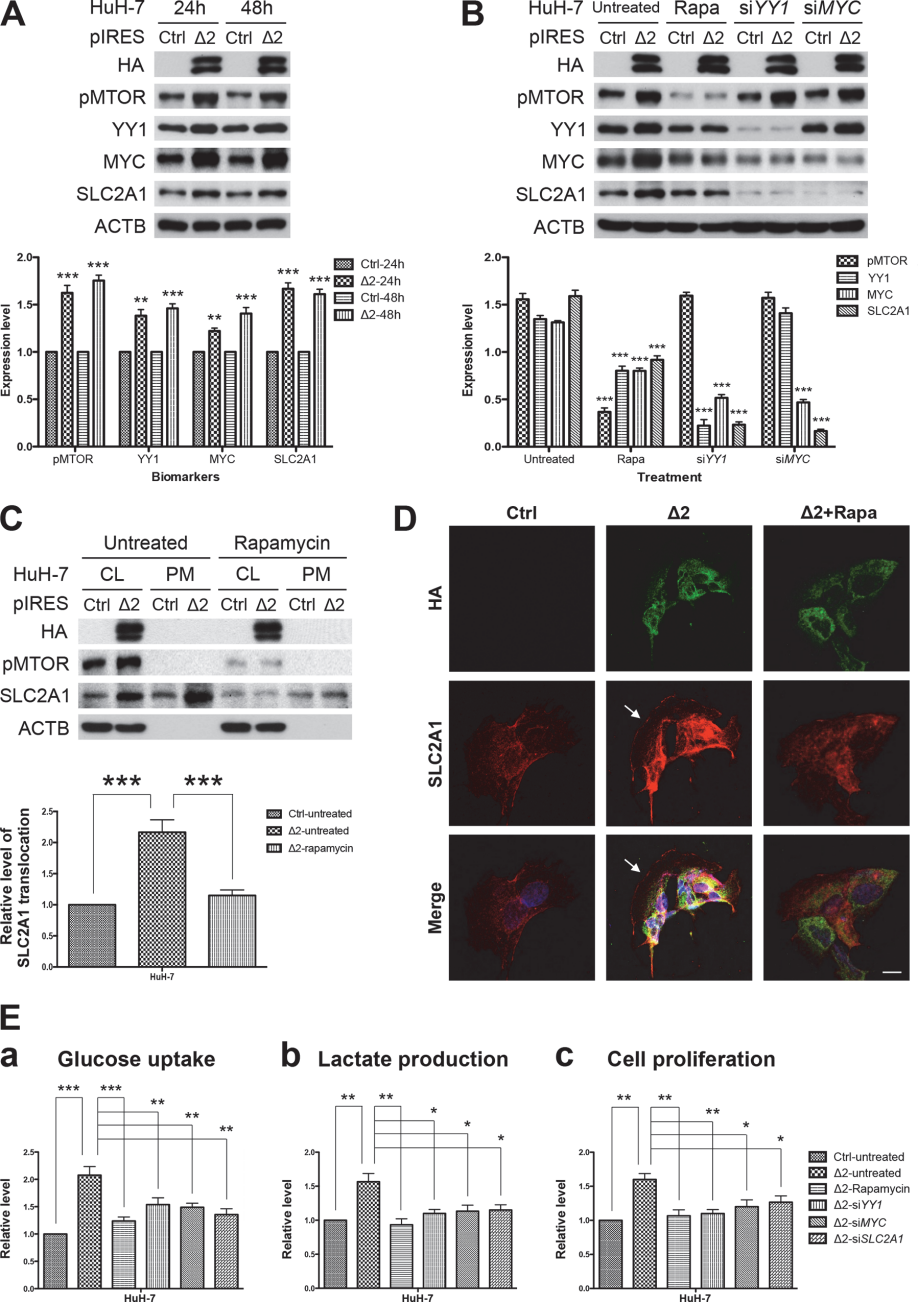
Pre-S2 mutant activated MTOR/YY1/MYC/SLC2A1 signaling cascade to promote SLC2A1 translocation, aerobic glycolysis, and growth advantages in HuH-7 cells: (A and B) HuH-7 cells were transfected with pre-S2 mutant or control plasmid (Ctrl). After 24 hours (h), cells were left untreated or treated with rapamycin (Rapa), *YY1* siRNA (si*YY1*) and *MYC* siRNA (si*MYC*) for another 24 hours, and analyzed by Western blot for the indicated biomarkers. (C) Western blots of the whole cell lysate fraction (CL) and the plasma membrane fraction (PM). SLC2A1 translocation represented the level of SLC2A1 in the PM fraction. (D) Confocal microscopy and IF staining of HA (green), SLC2A1 (red), and DAPI (blue). Arrows indicate the peripheral expression of SLC2A1. Original magnification, ×80. Scale bar, 20 μm. (E) For functional *in vitro* assays, HuH-7 cells transfected with pre-S2 mutant or control plasmid with or without further treatment were subjected to glucose uptake (a), lactate production (b), and cell proliferation (c) assays 48 hours after transfection. Data in each experiment were presented as relative values to the untreated control cells. All the transfection experiments were performed in triplicate and repeated at least three times independently.

To confirm that the MTOR/YY1/MYC/SLC2A1 signaling cascade was indeed induced by pre-S2 mutant, HuH-7 cells were transfected with pre-S2 mutant or control plasmid and then analyzed by Western blot assay. As shown in Fig. 3A, the expression of all the examined signaling molecules was increased at 24 and 48 hours post-transfection in pre-S2 mutant-transfected cells, as compared to the control cells. The upregulation of these signals by pre-S2 mutant was apparently mediated by MTOR, as the effect could be abolished by the MTOR inhibitor rapamycin (Fig. 3B). Also, selective knockdown of YY1 and MYC by siRNAs could sequentially diminish the elevated signals from upstream activators to downstream targets, even under the conditions of MTOR activation. Similar results were obtained from HepG2 cells (S2 Fig.). Taken together, these data suggest that pre-S2 mutant could upregulate SLC2A1 via the MTOR/YY1/MYC signaling. Considering that MYC is also a master regulator of cell cycle progression,[24] we further examined the candidate MYC-regulated cell cycle progression genes. The results revealed that cyclin A2 (CCNA2), cyclin E1 (CCNE1), and E2F transcription factor 2 (E2F2) were significantly increased in the transgenic HCCs and could be upregulated by pre-S2 mutant through MYC activation in HCC cells (S3 Fig.).

### Activation of MTOR signal cascade by pre-S2 mutant promoted SLC2A1 translocation, aerobic glycolysis, and growth advantages in HuH-7 cells

Since the functional site of which SLC2A1 mediated cellular glucose uptake is on the plasma membrane, we further assessed whether the level of SLC2A1 translocation to cell surface was increased by pre-S2 mutant in HuH-7 cells. As shown in Fig. 3C, when compared with the control cells, pre-S2 mutant-expressed cells showed significantly higher levels of SLC2A1 in the plasma membrane fraction, which could be reversed by rapamycin treatment. This MTOR-mediated SLC2A1 translocation pattern was further confirmed by confocal microscopy and IF staining, as indicated by the peripheral expression of SLC2A1 in pre-S2 mutant-expressed cells (Fig. 3D). Considering SLC2A1 as a key rate-limiting factor for aerobic glycolysis in cancer cells,[25] we next evaluated the effect of the MTOR signal cascade on aerobic glycolysis in HuH-7 cells. As shown in Fig. 3Ea and 3Eb, pre-S2 mutant transfections resulted in stimulation of glucose uptake and lactate production in comparison with the control cells, and this effect could be abrogated by inhibition of MTOR, YY1, MYC, and SLC2A1 signals. It has been proposed that aerobic glycolysis plays a fundamental role in supporting tumor proliferation.[12] To further characterize the role of this MTOR-induced glycolytic pathway in HCC cells, we performed functional *in vitro* assays with HuH-7 cells. As shown in Fig. 3Ec, pre-S2 mutant-transfected cells exhibited increased proliferation as compared to control cells. Following treatment of the transfected cells with inhibitors or siRNAs targeting to the signaling molecules significantly slowed the cell proliferation. Similar results were obtained with HepG2 cells (S4 Fig.). Collectively, these results suggested that pre-S2 mutant could stimulate aerobic glycolysis, which was required for tumorigenicity of HCC cells.

### Pre-S2 mutant activated the MTOR signal cascade through EIF4EBP1

**Fig 4:**
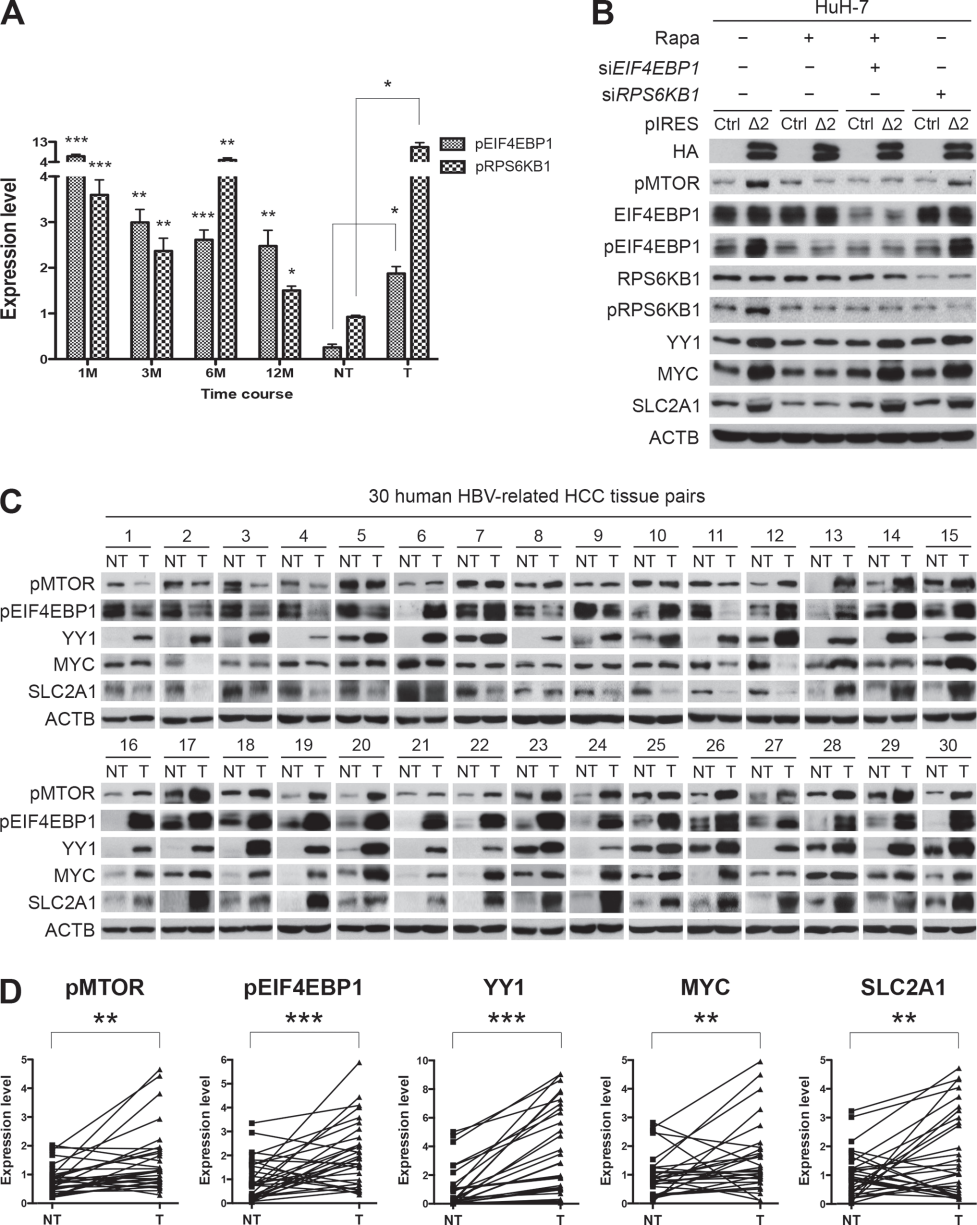
Activation of MTOR/YY1/MYC/SLC2A1 signaling by pre-S2 mutant was mediated by EIF4EBP1 and validated in human HBV-related HCCs: (A) Quantitative results of Western blots of pEIF4EBP1 and pRPS6KB1 were normalized by age-matched control livers. (B) The effect of *EIF4EBP1* and *RPS6KB1* siRNAs on MTOR signaling activation was determined by Western blot assay in HuH-7 cells. (C) Western blot analysis revealed enhanced expression of the indicated biomarkers in HBV-related HCCs at a level comparable to or even higher than that in the paired nontumorous livers. (D) The data were quantified and statistically analyzed.

A major function of MTOR is to promote protein synthesis by phosphorylating EIF4EBP1 and ribosomal protein S6 kinase, 70kDa, polypeptide 1 (RPS6KB1) [17]. In this study, the phosphorylation of EIF4EBP1 and RPS6KB1 was examined by Western blot assay and shown to increase throughout the entire period in pre-S2 mutant transgenic livers (Fig. 4A; raw data in S5A Fig.). To further clarify the effects, pre-S2 mutant-transfected HuH-7 cells were treated with rapamycin, *EIF4EBP1* or *RPS6KB1* siRNA. As shown in Fig. 4B, pre-S2 mutant-expressed cells exhibited increased levels of pMTOR, pEIF4EBP1, pRPS6KB1, YY1, MYC, and SLC2A1 signals, all of which could be abolished by MTOR inhibition. Knockdown of EIF4EBP1 could rescue the signaling activation under rapamycin treatment. However, knockdown of RPS6KB1 showed no effect on MTOR signaling activation. Similar results were obtained with HepG2 cells (S5B Fig.). Therefore, these data suggested that EIF4EBP1 is the mediator for the pre-S2 mutant-induced MTOR signal cascade.

### The MTOR/EIF4EBP1/YY1/MYC/SLC2A1 signaling was activated in human HBV-related HCCs

To ascertain whether MTOR/EIF4EBP1/YY1/MYC/SLC2A1 signaling is associated with human HBV-related hepatocarcinogenesis, Western blot analysis was performed on 30 pairs of HBV-related HCCs and adjacent nontumorous livers for the expression of components of the signaling pathway. As shown in Fig. 4C and 4D, we demonstrated that pMTOR, pEIF4EBP1, YY1, MYC, and SLC2A1 were consistently and significantly expressed at higher levels in HCCs than in the paired nontumorous livers in 18 of 30 tissue pairs (cases 13–30). In summary, the data support the essential role of the glycolysis-associated MTOR signal cascade in HBV tumorigenesis.

### The MTOR/EIF4EBP1/YY1/MYC/SLC2A1 signaling mediated the chemopreventive effect of combined resveratrol and silymarin product on tumor growth

**Fig 5:**
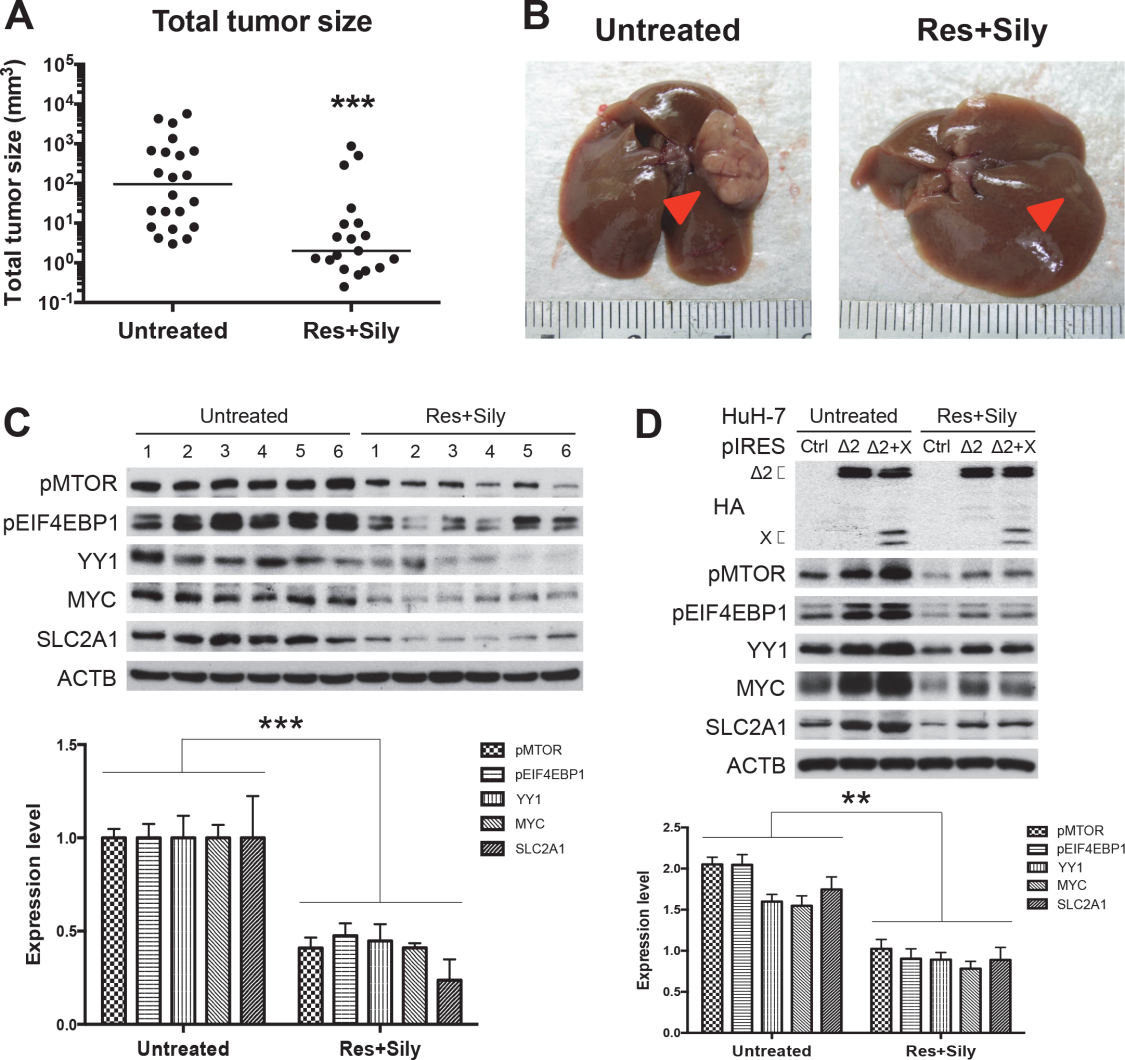
The MTOR/EIF4EBP1/YY1/MYC/SLC2A1 signaling mediated the chemopreventive effect of combined resveratrol and silymarin product on tumor growth: Total tumor size (A) and gross view of representative HCCs (B) with or without treatment of resveratrol (Res) and silymarin (Sily). Arrows indicate tumors. (C and D) Western blot analysis revealed a lower expression level of MTOR signal cascade in the treated cells and mice group (six smallest tumor adjacent tissues) than the untreated cells and mice group (six biggest tumor adjacent tissues). Quantitative results by coexpression of pre-S2 mutant and X proteins were relative to the untreated control cells and mice.

Resveratrol and silymarin are naturally occurring compounds that have anticarcinogenic effects on various types of cancers [26,27]. In the model of chemopreventive study in transgenic mice harboring both HBV pre-S2 mutant and X proteins, the combined resveratrol and silymarin product has been shown to inhibit HCC growth [28]. As shown in Fig. 5A and 5B, the combined product of resveratrol and silymarin could significantly decrease the transgenic tumor size as compared with the untreated group. Western blot analysis performed on the expression profiles of the MTOR signal cascade in the tumor adjacent tissues revealed that the treated group expressed a lower level of MTOR/EIF4EBP1/YY1/MYC/SLC2A1 signaling than the untreated group (Fig. 5C). We further examined the *in vitro* effect of resveratrol and silymarin on MTOR/EIF4EBP1/YY1/MYC/SLC2A1 signaling cascade in HBV tumorigenesis. Consistent with pre-S2 mutant alone, we observed that HuH-7 cells (Fig. 5D) and HepG2 cells (S6 Fig.) expressing both pre-S2 mutant and X proteins showed activation of the MTOR signal cascade, which could be inhibited by the combined resveratrol and silymarin treatment.

## Discussion

**Fig 6:**
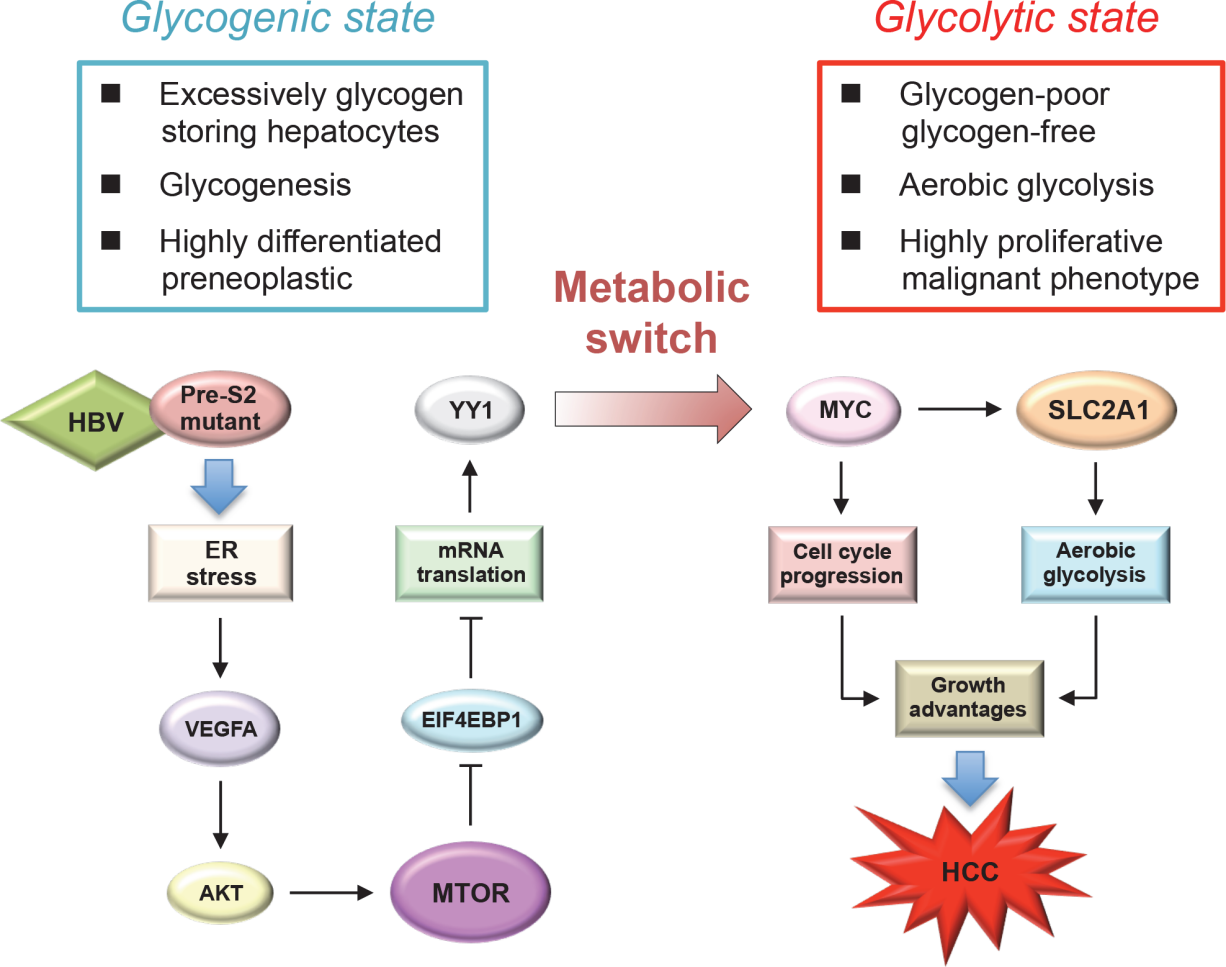
Schematic model for the upregulation of aerobic glycolysis by pre-S2 mutant in HBV tumorigenesis: Through the induction of ER stress-dependent VEGFA/AKT signaling, pre-S2 mutant activates MTOR signal, which then increases YY1 expression through the EIF4EBP1-mediated translational control. Upon the activation of MYC and SLC2A1 signals by YY1 at the advanced stage of HCC tumorigenesis, hepatocytes may undergo a metabolic switch toward an increase in aerobic glycolysis. The combined effects of aerobic glycolysis and cell cycle progression induced by MYC and SLC2A1 may contribute to growth advantages of hepatocytes and HCC development.

This study demonstrated the contributing role of HBV pre-S2 mutant in metabolic disturbances of HBV-related HCC development mediated via ER stress-induced, MTOR-dependent glycolytic signal cascade. Our findings, together with other previous studies [19,29], support the important role of MTOR signaling as a molecular regulator linking metabolic disorder and cancer in chronic HBV infection. In this study, we demonstrated that pre-S2 mutant could stimulate aerobic glycolysis through activation of the MTOR/YY1/MYC signaling to upregulate SLC2A1. Upon the activation of SLC2A1 at the advanced stage of tumorigenesis, hepatocytes underwent a metabolic switch from the glycogen-storage state toward increased aerobic glycolysis. SLC2A1 belongs to the class I of SLC2A family of proteins (SLC2A1-4) whose expression level usually correlates with the rate of cellular glucose metabolism [25]. The increased SLC2A1 expression in HCC does not only indicate an increased utilization of energy but can also directly cause tumorigenesis [30]. Therefore, our findings suggest that pre-S2 mutant may promote tumorigenesis by sustaining high activation rates of aerobic glycolysis through the MTOR signal cascade, as summarized in Fig. 6.

One novel finding in this study is the upregulation of MYC by pre-S2 mutant-activated MTOR signal through YY1 to activate SLC2A1. In this pre-S2 mutant transgenic mouse model, we observed that MTOR/YY1 signaling was activated throughout the entire period in transgenic liver tissues, but MYC was overexpressed only until HCC developed. YY1 has been shown to regulate MYC expression in certain scenarios. In normal cells, YY1 forms a ternary complex with the transcriptional corepressors E1A binding protein p300 and histone deacetylase 3 to repress *MYC* transcription [31]. In transformed cells, YY1 may instead activate *MYC* promoter by recruiting the transcriptional coactivator and histone acetyltransferase CREB binding protein [32]. This proposed model for YY1-mediated transcriptional regulation of MYC may possibly explain the delayed overexpression of MYC in pre-S2 mutant transgenic HCCs. As a critical regulator of cell proliferation, MYC regulates a number of genes involved in a variety of biological processes, including glycolysis, cell cycle, apoptosis, and cell adhesion [24]. However, which MYC target genes contribute to HBV-related tumorigenesis remains poorly defined. In this report, we demonstrated that the glycolytic gene *SLC2A1* and cell cycle progression genes *CCNA2*, *CCNE1*, and *E2F2* were upregulated by MYC through pre-S2 mutant-induced MTOR activation, providing a candidate signaling pathway driven by MYC in HBV-related HCCs.

In this study, we further clarified that MTOR-mediated YY1 upregulation by pre-S2 mutant was mediated through phosphorylating its downstream effector EIF4EBP1, a repressor of mRNA translation. As a master regulator of protein synthesis, recent studies have uncovered that MTOR regulates the selective translation of mRNAs with 5’ terminal oligopyrimidine (TOP) and TOP-like motifs through the EIF4EBP1-dependent translational control [33]. These reports support our findings, since *YY1* mRNA can be classified into the subset of TOP-like mRNAs according to the defined criteria [34], and provide a molecular explanation for the translational upregulation of YY1 by MTOR. Although YY1 has been reported to be highly expressed in HCC [22], its exact role in HBV-related HCC development remains largely unclear. In this study, we demonstrated that YY1 overexpression by pre-S2 mutant-induced MTOR activation could stimulate aerobic glycolysis through MYC to upregulate SLC2A1, proposing a novel oncogenic role of YY1 in HCC development.

In this study, we demonstrated that SLC2A1 was overexpressed in pre-S2 mutant-expressed HCC cell lines and human HBV-related HCC tissues. In addition to SLC2A1, the effect of pre-S2 mutant on the expression of other class I SLC2A proteins was examined. As shown in S7 Fig., we observed no significant difference in SLC2A2, SLC2A3, and SLC2A4 expression between the pre-S2 mutant transgenic HCC and nontumorous liver tissues. Also, pre-S2 mutant showed no effect on the expression of these three SLC2A proteins in HCC cells.

The chemopreventive effects of resveratrol and silymarin compounds on liver pathology and HCC have been proposed [35,36]. In this study, we demonstrated that MTOR/EIF4EBP1/YY1/MYC/SLC2A1 signaling cascade in pre-S2 mutant-mediated hepatocarcinogenesis could be inhibited by the combined treatment of resveratrol and silymarin. The results suggest that the downregulation of the MTOR signal cascade may be an important mechanism for the chemopreventive effect of resveratrol and silymarin on HCC development. The MTOR inhibitor rapamycin has been shown to synergistically enhance the resveratrol-induced apoptotic cell death [37].

In conclusion, this study demonstrates that the pre-S2 mutant-induced MTOR signal cascade plays a crucial role in driving the metabolic alterations toward increased aerobic glycolysis through the activation of MYC and SLC2A1 signals in HBV-associated tumorigenesis. Targeting the MTOR/EIF4EBP1/YY1/MYC/SLC2A1 signaling pathway may provide avenues for HCC therapy.

## Supporting Information

S1 Fig *Myc* and *Slc2a1* were identified as candidate genes regulating glycolysis in pre-S2 mutant transgenic HCCs: Microarray expression profiles of selected YY1-activated oncogenes (A) and MYC-activated glycolytic genes (B) in different months (M) of pre-S2 mutant transgenic livers, nontumorous livers (NT), and tumors (T). All values were shown as fold changes relative to the data of age-matched non-transgenic livers. Numbers on a gray background represent ≥1.5-fold increase. (C) Transcript levels of *Myc* and *Slc2a1* in each stage of transgenic livers relative to the control livers were measured by real-time PCR. Abbreviations are: *Myc*, myelocytomatosis oncogene; *Ptgs2*, prostaglandin-endoperoxide synthase 2; *Tgfb1*, transforming growth factor, beta 1; *Hspa5*, heat shock 70kDa protein 5; *Erbb2*, v-erb-b2 avian erythroblastic leukemia viral oncogene homolog 2; *Snai1*, snail family zinc finger 1; *Npm1*, nucleophosmin; *Otx2*, orthodenticle homeobox 2; *Msx2*, msh homeobox 2; Slc2a1, solute carrier family 2 (facilitated glucose transporter), member 1; *Ldha*, lactate dehydrogenase A; *Hk2*, hexokinase 2; *Pfkm*, phosphofructokinase, muscle; *Eno1*, enolase 1.
(TIF)

S2 Fig Pre-S2 mutant activated MTOR/YY1/MYC/SLC2A1 signaling cascade in HepG2 cells: (A and B) HepG2 cells were transfected with pre-S2 mutant or control plasmid (Ctrl). After 24 hours (h), cells were left untreated or treated with rapamycin (Rapa), *YY1* siRNA (si*YY1*) and *MYC* siRNA (si*MYC*) for another 24 hours, and analyzed by Western blot for the indicated biomarkers. Data in each experiment were presented as relative values to the untreated control cells.
(TIF)

S3 Fig Pre-S2 mutant upregulated CCNA2, CCNE1, and E2F2 through MYC activation in transgenic livers, HuH-7 cells, and HepG2 cells: Expression profiles of the selected cell cycle genes in pre-S2 mutant transgenic livers (A and B), HuH-7 cells (C), and HepG2 cells (D) were determined by Western blot assay. Quantitative results were normalized by the age-matched control livers or the untreated cells.
(TIF)

S4 Fig Activation of MTOR/YY1/MYC/SLC2A1 signaling by pre-S2 mutant promoted SLC2A1 translocation, aerobic glycolysis, and growth advantages in HepG2 cells: (A and B) Western blots of the whole cell lysate fraction (CL) and the plasma membrane fraction (PM) of pre-S2 mutant- or control plasmid-transfected HepG2 cells. SLC2A1 translocation represented the level of SLC2A1 in the PM fraction. (C) For functional *in vitro* assays, HepG2 cells transfected with pre-S2 mutant or control plasmid with or without further treatment were subjected to glucose uptake (a), lactate production (b), and cell proliferation (c) assays. Data in each experiment were presented as relative values to the untreated control cells.
(TIF)

S5 Fig Pre-S2 mutant activated the MTOR signal cascade through EIF4EBP1: (A) Expression profiles of pEIF4EBP1 and pRPS6KB1 in pre-S2 mutant and non-transgenic livers were established by Western blots. (B) The effect of *EIF4EBP1* and *RPS6KB1* siRNAs on MTOR signaling activation was determined by Western blot assay in HepG2 cells.
(TIF)

S6 Fig Combined resveratrol and silymarin treatment inhibited the MTOR signal cascade in HepG2 cells overexpressing pre-S2 mutant and X proteins: Western blot assay was performed to examine the expression of the indicated biomarkers. Quantitative results by coexpression of pre-S2 mutant and X proteins were relative to the untreated control cells.
(TIF)

S7 Fig Pre-S2 mutant showed no effect on SLC2A2, SLC2A3, and SLC2A4 expression: The expression profiles of SLC2A2, SLC2A3, and SLC2A4 in pre-S2 mutant transgenic livers (A), HuH-7 cells (B), and HepG2 cells (C) were examined by Western blot assay. (TIF)

S1 Table Primers for real-time PCR. (DOC)
