# Supplementary figures and images for: Hepatitis B Virus Pre-S2 Mutant Induces Aerobic Glycolysis through Mammalian Target of Rapamycin Signal Cascade
Source: PLoS One. 2015 Apr 24;10(4):e0122373. doi: 10.1371/journal.pone.0122373 (PMC4409318; doi:10.1371/journal.pone.0122373)

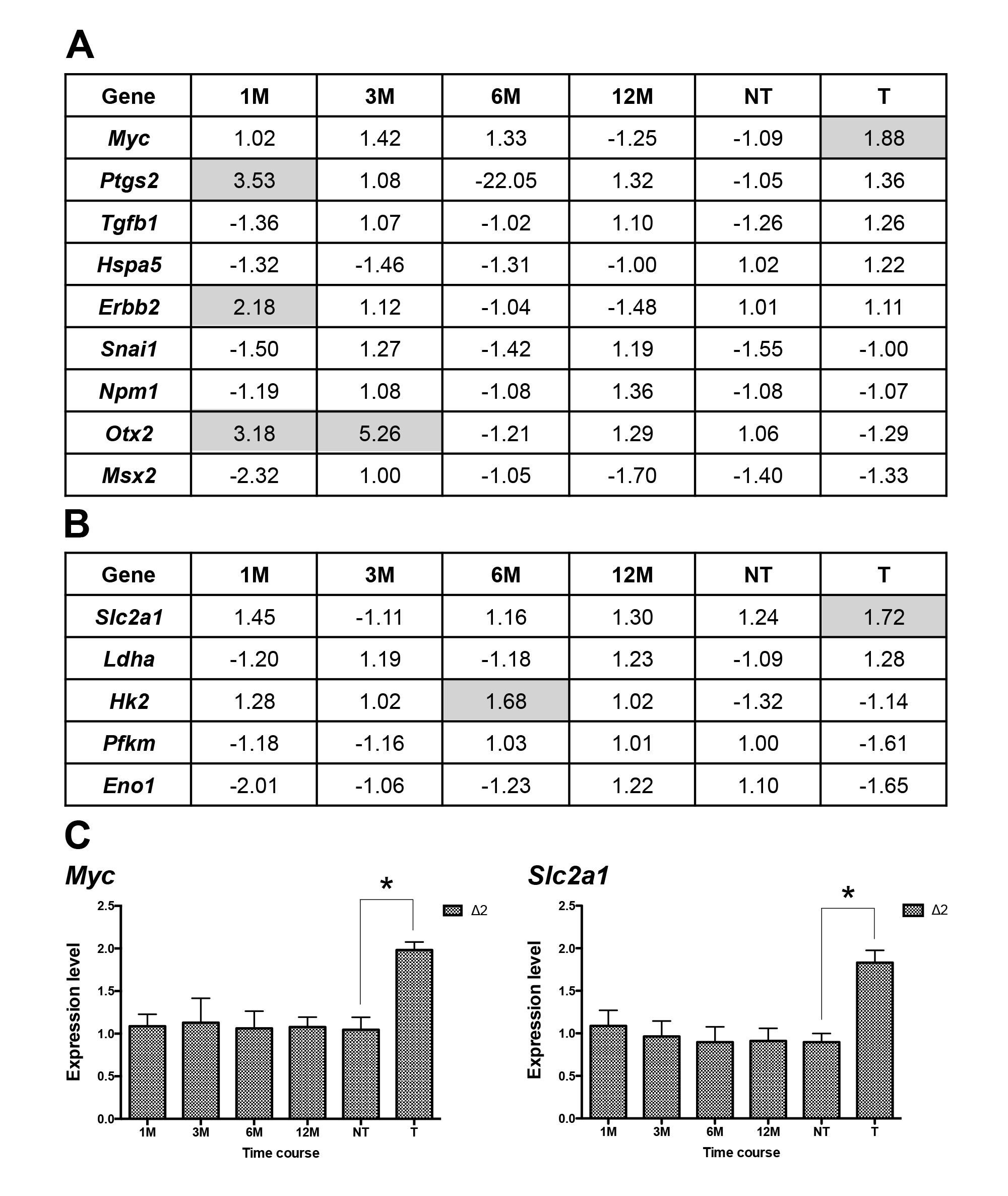

Supplement: S1 Fig — All values were shown as fold changes relative to the data of age-matched non-transgenic livers. Numbers on a gray background represent ≥1.5-fold increase. (C) Transcript levels of Myc and Slc2a1 in each stage of transgenic livers relative to the control livers were measured by real-time PCR. Abbreviations are: Myc, myelocytomatosis oncogene; Ptgs2, prostaglandin-endoperoxide synthase 2; Tgfb1, transforming growth factor, beta 1; Hspa5, heat shock 70kDa protein 5; Erbb2, v-erb-b2 avian erythroblastic leukemia viral oncogene homolog 2; Snai1, snail family zinc finger 1; Npm1, nucleophosmin; Otx2, orthodenticle homeobox 2; Msx2, msh homeobox 2; Slc2a1, solute carrier family 2 (facilitated glucose transporter), member 1; Ldha, lactate dehydrogenase A; Hk2, hexokinase 2; Pfkm, phosphofructokinase, muscle; Eno1, enolase 1. (TIF) [file pone.0122373.s001.tif]

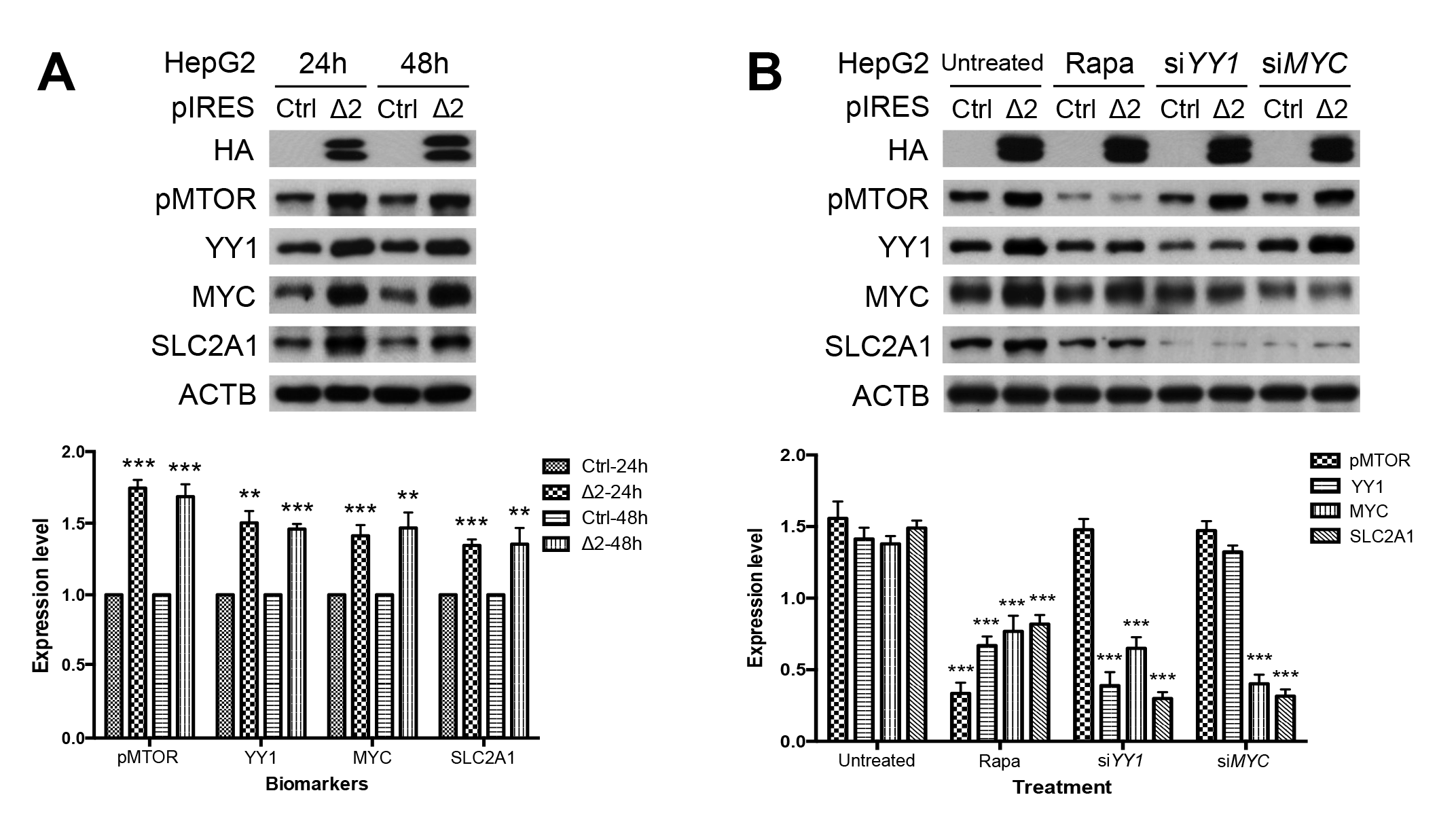

Supplement: S2 Fig — After 24 hours (h), cells were left untreated or treated with rapamycin (Rapa), YY1 siRNA (siYY1) and MYC siRNA (siMYC) for another 24 hours, and analyzed by Western blot for the indicated biomarkers. Data in each experiment were presented as relative values to the untreated control cells. (TIF) [file pone.0122373.s002.tif]

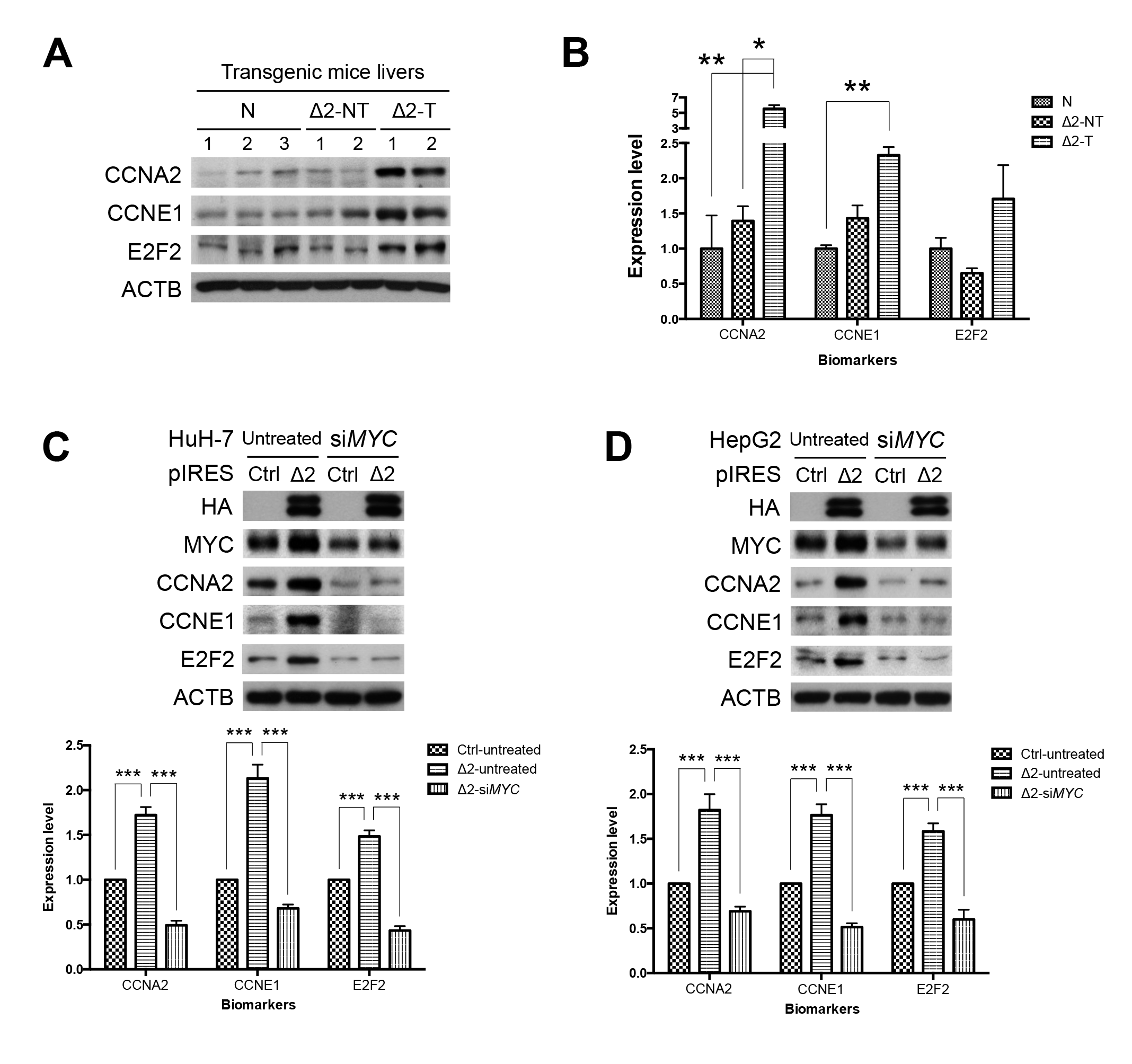

Supplement: S3 Fig — Quantitative results were normalized by the age-matched control livers or the untreated cells. (TIF) [file pone.0122373.s003.tif]

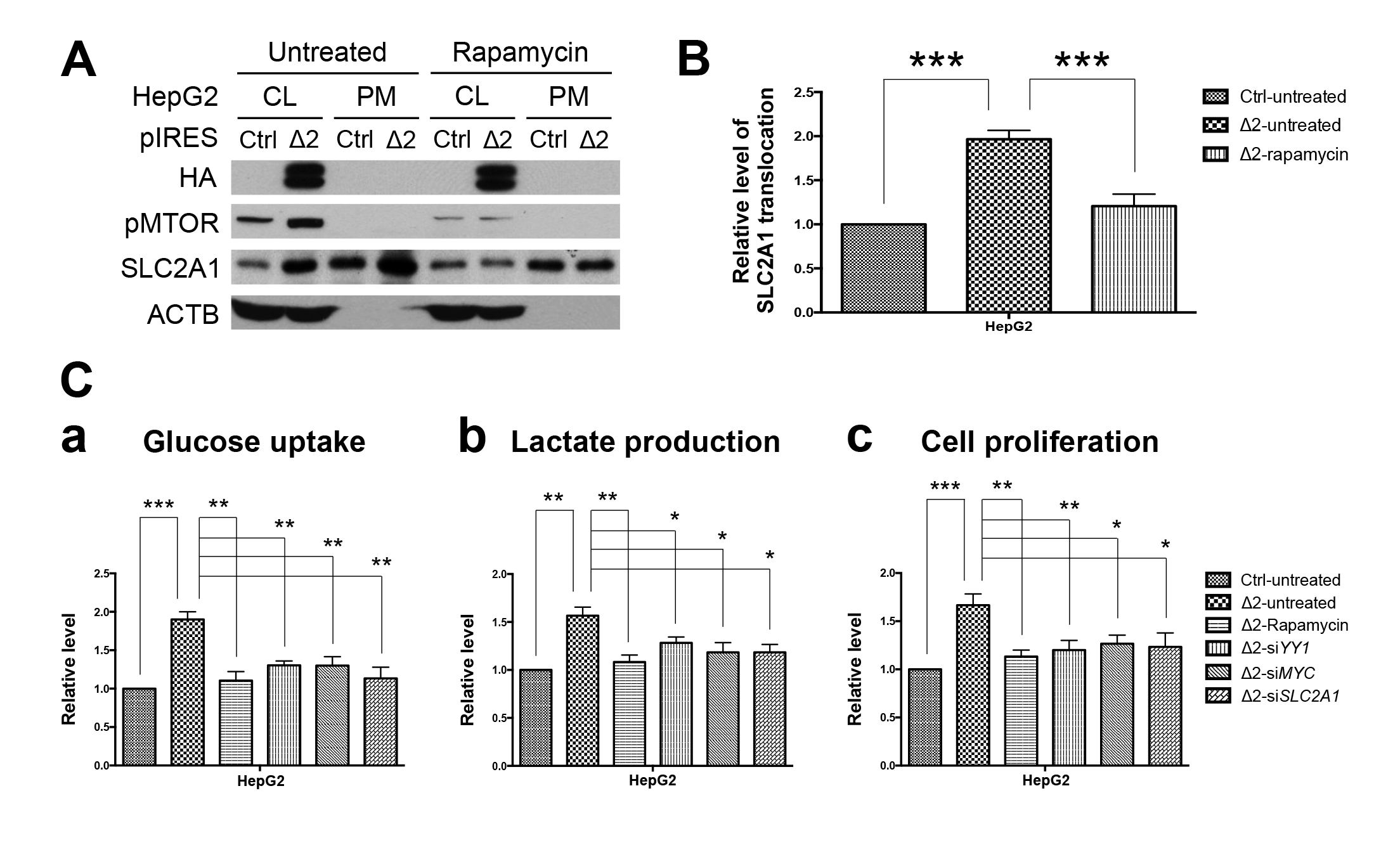

Supplement: S4 Fig — SLC2A1 translocation represented the level of SLC2A1 in the PM fraction. (C) For functional in vitro assays, HepG2 cells transfected with pre-S2 mutant or control plasmid with or without further treatment were subjected to glucose uptake (a), lactate production (b), and cell proliferation (c) assays. Data in each experiment were presented as relative values to the untreated control cells. (TIF) [file pone.0122373.s004.tif]

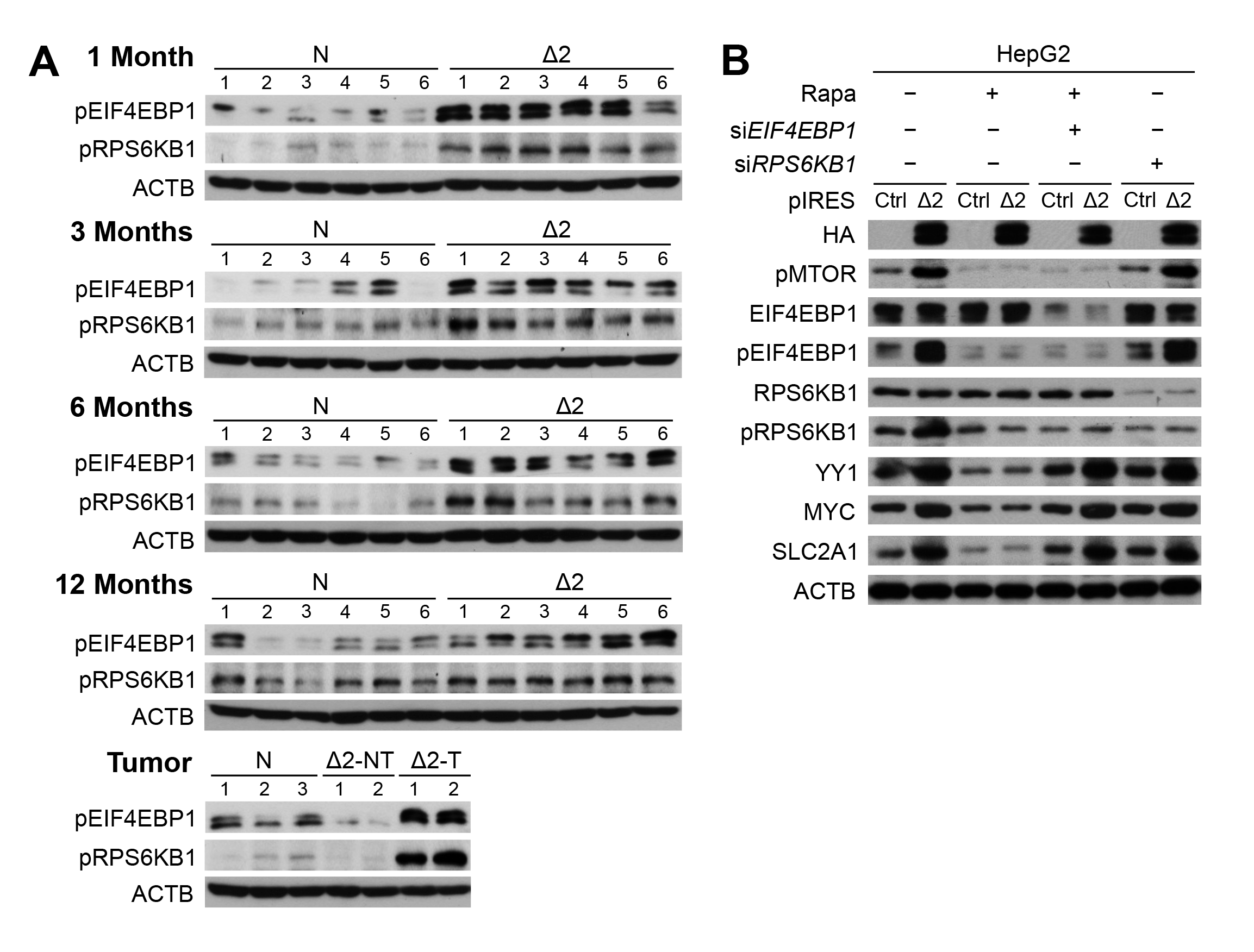

Supplement: S5 Fig — (B) The effect of EIF4EBP1 and RPS6KB1 siRNAs on MTOR signaling activation was determined by Western blot assay in HepG2 cells. (TIF) [file pone.0122373.s005.tif]

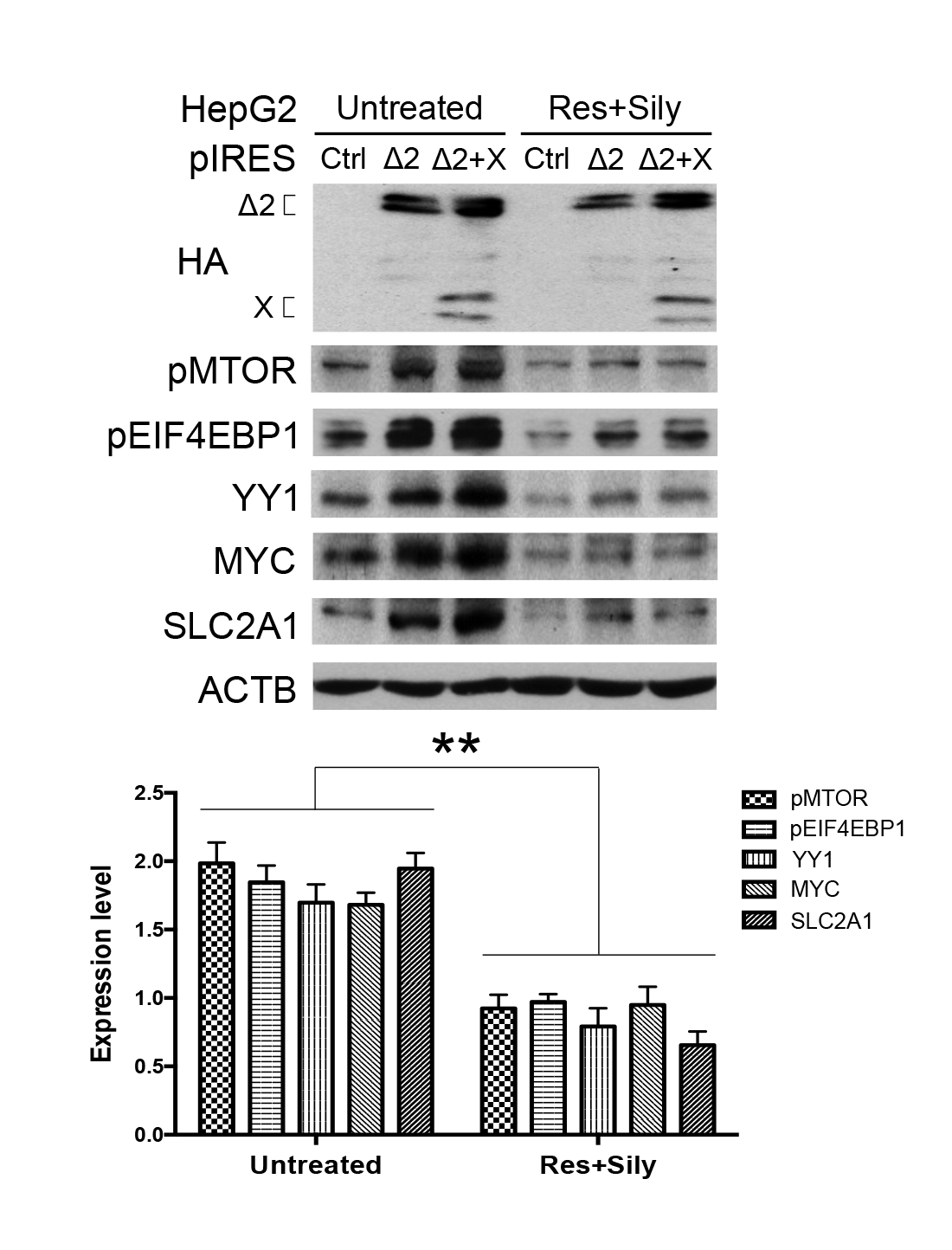

Supplement: S6 Fig — Quantitative results by coexpression of pre-S2 mutant and X proteins were relative to the untreated control cells. (TIF) [file pone.0122373.s006.tif]

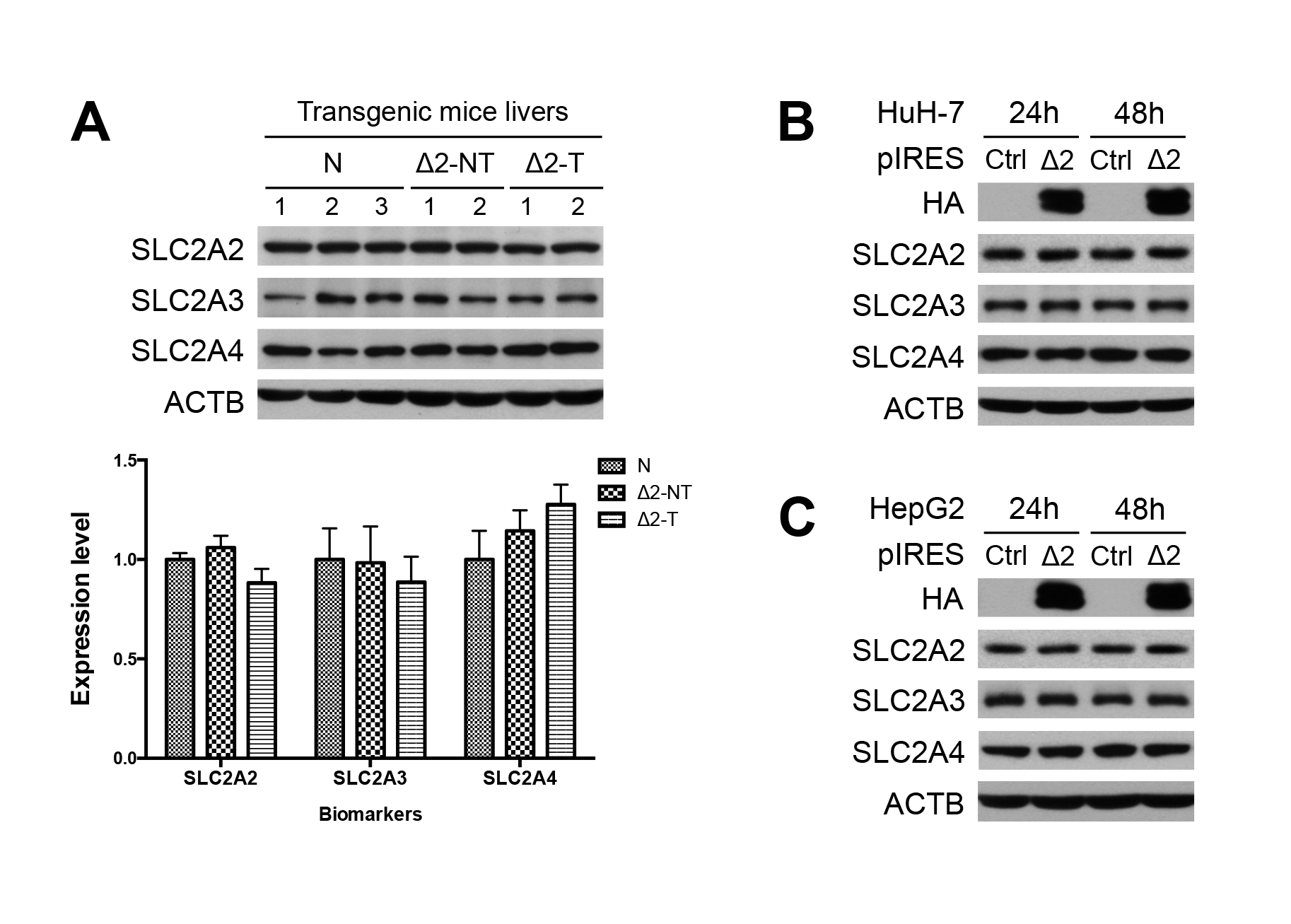

Supplement: S7 Fig — (TIF) [file pone.0122373.s007.tif]
