# Supplementary material for: Hepatitis B Virus Pre-S2 Mutant Induces Aerobic Glycolysis through Mammalian Target of Rapamycin Signal Cascade
Source: PLoS One. 2015 Apr 24;10(4):e0122373. doi: 10.1371/journal.pone.0122373 (PMC4409318; doi:10.1371/journal.pone.0122373)
Supplement: S1 Table — (DOC) [file pone.0122373.s008.doc]

**S1 Table. Primers for real-time PCR**

| **Primer** | **Sequence (5’ to 3’)** |
| --- | --- |
| *Myc* | (Forward)  CCTAGTGCTGCATGAGGAGA  (Reverse)  TCCACAGACACCACATCAATTT  (Universal ProbeLibrary probe)  number 77 |
| *Slc2a1* | (Forward)  GACCCTGCACCTCATTGG  (Reverse)  GATGCTCAGATAGGACATCCAAG  (Universal ProbeLibrary probe)  number 99 |
|  *Actb* | (Forward)  CTAAGGCCAACCGTGAAAAG  (Reverse)  ACCAGAGGCATACAGGGACA  (Universal ProbeLibrary probe)  number 64 |

Abbreviations are: *Myc*, myelocytomatosis oncogene; *Slc2a1*, solute carrier family 2 (facilitated glucose transporter), member 1; *Actb*, actin, beta.
